# Trends in incidence of cervical cancer in England, 1985-2019

**DOI:** 10.1093/eurpub/ckac131.140

**Published:** 2022-10-25

**Authors:** L Fox, P Bannister, A Memon

**Affiliations:** Department of Primary Care and Public Health, Brighton and Sussex Medical School, Brighton, UK

## Abstract

**Background:**

Worldwide, cervical cancer is the 4th most common cancer in women. The highest incidence is observed in Africa (25.6/100,000) and the lowest in North America (6.1/100,000). Over 99% of cases are caused by human papillomavirus (HPV). In the UK, HPV vaccination has been offered to school children aged 12-13 years since 2008. We conducted a retrospective population-based cohort study to examine whether there have been changes in the incidence of cervical cancer in England during the past four decades.

**Methods:**

Individual level data for women diagnosed with cervical cancer in England during 1985-2019 were obtained from the Office for National Statistics/Public Health England. Average annual incidence rates were calculated by two age categories (0-49, 50+ years) and all ages combined during the seven five-year time periods (1985-89 to 2015-19). The percentage change in incidence was calculated as change in the average annual incidence rate from the first (1985-89) to the last time period (2015-19). Index of Multiple Deprivation (IMD) quintiles (2015-19) were examined to determine the social gradient of the disease.

**Results:**

During the 35-year study period, a total of 100,303 women with cervical cancer were registered in England. In women aged 0-49 years, the average annual incidence rates declined by about 20% (from 11.6/100,000 in 1985-89 to 9.3/100,000 in 2015-19), and in women aged 50+ years, the rates declined by about 64% (from 26.1/100,000 in 1985-89 to 9.5/100,000 in 2015-19). At all ages combined, the rate declined by 43%. With regard to the social gradient, about half of the cases occurred in women in the most deprived quintiles.

**Conclusions:**

There has been a steady decline in the incidence of cervical cancer in England over the past four decades. These findings are consistent with reports from other Western countries. The finding of relatively increased risk of cervical cancer among women from most deprived communities needs further investigation.

**Key messages:**

• Considering that over 99% of cervical cancers are potentially preventable, there is an urgent need to support low- and middle-income countries to roll out HPV vaccination programme.

• The difference in risk of cervical cancer by social gradient highlights the importance of reducing health and social inequalities.

